# Effects of Exosomes Derived from Adipose-Derived Mesenchymal Stem Cells on Pyroptosis and Regeneration of Injured Liver

**DOI:** 10.3390/ijms232012065

**Published:** 2022-10-11

**Authors:** Chenxi Piao, Jinfang Sang, Zhipeng Kou, Yue Wang, Tao Liu, Xiangyu Lu, Zhihui Jiao, Hongbin Wang

**Affiliations:** 1 College of Veterinary Medicine, Northeast Agricultural University, Harbin 150030, China; 2 College of Wildlife and Protected Area, Northeast Forestry University, Harbin 150030, China

**Keywords:** hepatic ischemia–reperfusion injury, ADSCs-Exo, pyroptosis, liver regeneration, rats

## Abstract

Although accumulating evidence indicates that exosomes have a positive therapeutic effect on hepatic ischemia–reperfusion injury (HIRI), studies focusing on the alleviation of liver injury by exosomes derived from adipose-derived mesenchymal stem cells (ADSCs-Exo) based on the inhibition of cell pyroptosis have not yet been reported. Exosomes contain different kinds of biologically active substances such as proteins, lipids, mRNAs, miRNAs, and signaling molecules. These molecules are widely involved in cell–cell communication, cell signal transmission, proliferation, migration, and apoptosis. Therefore, we investigated the positive effects exerted by ADSCs-Exo after hepatic ischemia–reperfusion with partial resection injury in rats. In this study, we found that the post-operative tail vein injection of ADSCs-Exo could effectively inhibit the expression of pyroptosis-related factors such as NLRP3, ASC, caspase-1, and GSDMD-N, and promote the expression of regeneration-related factors such as Cyclin D1 and VEGF. Moreover, we found that the above cellular activities were associated with the NF-κB and Wnt/β-catenin signaling pathways. According to the results, ADSCs and ADSCs-Exo can reduce pyroptosis in the injured liver and promote the expression of those factors related to liver regeneration, while they can inhibit the NF-κB pathway and activate the Wnt/β-catenin pathway. However, although adipose-derived mesenchymal stem cell (ADSC) transplantation can reduce liver injury, it leads to a significant increase in the pyroptosis-related protein GSDMD-N expression. In conclusion, our study shows that ADSCs-Exo has unique advantages and significance as a cell-free therapy to replace stem cells and still has a broad research prospect in the clinical diagnosis and treatment of liver injuries.

## 1. Introduction

Liver operations, such as partial hepatectomy or liver transplantation, block the blood flow to the liver for some time. However, when the blood flow is recovered, it causes liver ischemia, which causes further damage. Hepatic ischemia–reperfusion injury (HIRI) is one of the important causes of liver injury or liver failure after liver surgery. Massive cell death, severe inflammation, and oxidative stress are the typical pathological characteristics of tissues undergoing ischemia–reperfusion injury.

Pyroptosis, also known as inflammatory necrosis, is different from apoptosis and necrosis. It depends on the inflammasome and is accompanied by an inflammatory response. The inflammasome is a kind of polyprotein complex that is an important component in the process of cell death. It can be divided into two categories based on the receptor proteins, namely AIM2-like receptors (ALRs) and nucleotide-binding oligomerization domain-like receptors (NLRs). As cellular receptors, NLRs play important roles in innate immunity and can mediate the mitogen-activated protein kinase (MAPK) and nuclear transcription factor κB (NF-κB) signaling pathways. Other intracellular receptors such as NLRP1 and NLRP3 lead to the assembly of inflammasome complexes and activate caspase-1, IL-1β, IL-18, and other inflammatory factors. Many studies have shown that the activation of NLRP3 is important for inducing HIRI [1,2]. In the HIRI model of diabetic mice, it was proved that the NLRP3 inflammasome was activated by increased ROS, which promoted an increase in cell death. In contrast, CY09, an NLRP3 inhibitor, could alleviate liver injury [3]. Hippo signaling controls the activation of NLRP3 in mesenchymal stem cell (MSC)-induced immune regulation and is a key regulator that drives the inflammatory response in HIRI [4].

NF-κB plays an important regulatory role in inflammatory responses, including the p50/p65 heterodimer. In its normal state, NF-κB is bound to IκB and is in an inactive form. When cells are stimulated, IκB is phosphorylated and releases NF-κB in an activated form. In the active form of NF-κB, the nuclear localization signal of p50 is leaked, and it is rapidly transferred to the nucleus, where p65 recognizes specific DNA sequences and enhances the expression of inflammatory factors. The overexpression of a variety of inflammatory factors further activates NF-κB, thus amplifying the cascade of the inflammatory response [5]. Studies have shown that a large amount of ROS activates NF-κB and promotes the release of inflammatory cytokines. This activates the NLRP3 inflammasome and increases the IL-1β and IL-18 secretion, which further promotes the death of inflammatory cells, thereby aggravating tissue damage [6].

Exosomes are tiny vesicles with bilayer membrane structures of only 30–150 nm in diameter and are formed by eukaryotic cells through a series of regulatory processes such as “endocytosis–fusion–efflux” and can be secreted into the extracellular space [7]. Adipose-derived mesenchymal stem cells (ADSCs) are highly promising for cell-based regenerative therapy given their abundance, ease of harvest, and non-immunogenicity. The exosomes derived from ADSCs (ADSCs-Exo) have been shown to be effective in treating rat liver injury. They can restore liver function and structure by reducing mitochondrial damage and cell apoptosis [8]. However, the effect of ADSCs-Exo on cell pyroptosis and regeneration in rat liver ischemia–reperfusion combined with partial resection injury has not yet been reported.

## 2. Results

### 2.1. ADSCs-Exo Attenuate Liver Pyroptosis

**Figure 1:**
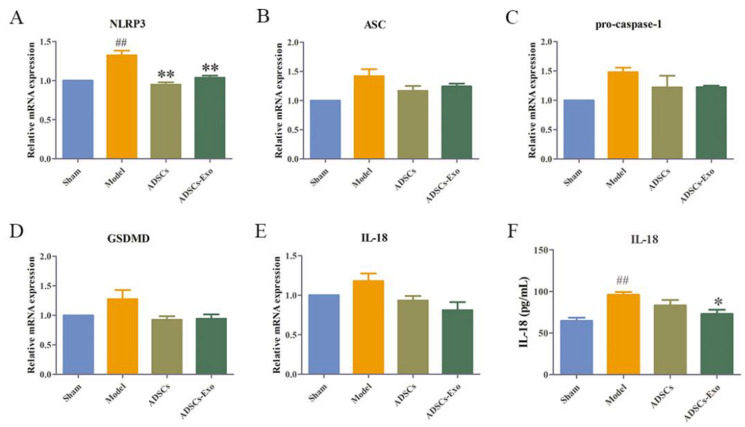
ADSCs-Exo decreased the gene expression of pyroptosis in the liver. Level of NLRP3 (**A**), ASC (**B**), pro-caspase-1 (**C**), GSDMD (**D**), and IL-18 (**E**) in the liver in different groups. Level of IL-18 protein in the level in different groups (**F**). The data are expressed as mean ± SD. ^##^ *p* < 0.01, versus the Sham group. * *p* < 0.05 and ** *p* < 0.01, versus the Model group.

**Figure 2:**
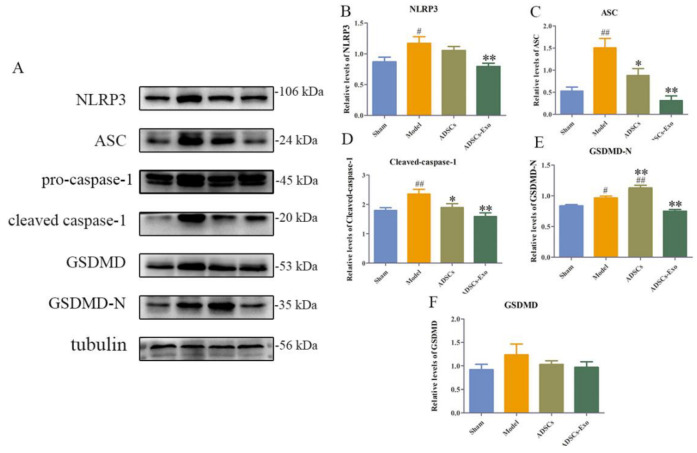
ADSCs-Exo decreased the protein expression of pyroptosis in the liver. Representative Western blot analysis of NLRP3, ASC, pro-caspase-1, cleaved caspase-1, GSDMD, and GSDMD-N (**A**). Quantification of NLRP3, ASC, cleaved caspase-1, GSDMD-N, and GSDMD (**B**–**F**). The data are expressed as mean ± SD. ^#^ *p* < 0.05 and ^##^ *p* < 0.01, versus the Sham group. * *p* < 0.05 and ** *p* < 0.01, versus the Model group.

Ischemia and reperfusion combined with hepatectomy markedly increased the expression levels of NLRP3, ASC, cleaved caspase-1, GSDMD-N, and GSDMD protein. ADSC transplantation significantly reduced ASC and cleaved caspase-1 but remarkably increased the GSDMD-N protein expression. However, ADSCs-Exo significantly reduced the expression of cell-pyroptosis-related proteins such as NLRP3, ASC, cleaved-caspase-1, GSDMD-N, and IL-18 (Figure 1F and Figure 2). Similar results were observed in the mRNA expression profiles of liver tissue after injury and intervention (Figure 1A–E).

### 2.2. ADSCs-Exo Inhibited p65 Expression and Phosphorylation of p65

**Figure 3:**
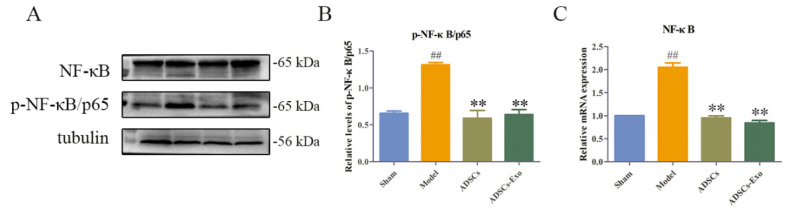
Western blot and qRT-PCR analysis of NF-κB-related protein and gene expression in the liver. Western blot analysis of p-NF-κB (**A**). Quantification of p-NF-κB (**B**). mRNA level of NF-κB in the liver in different groups (**C**). The data are expressed as mean ± SD. ^##^ *p* < 0.01, versus the Sham group. ** *p* < 0.01, versus the Model group.

**Figure 4:**
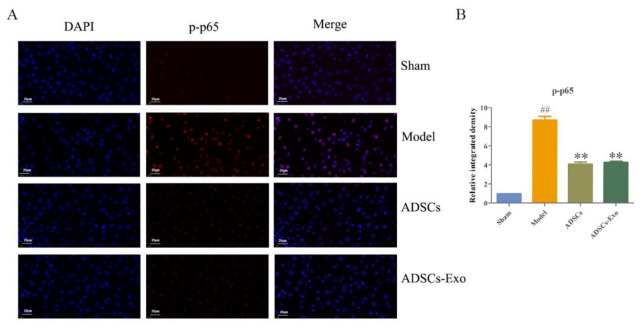
The protein expression of p-p65 in the liver. Representative immunostaining images of p-p65 in the different groups (**A**). Scale bar 100 μm. Quantification of the fraction of p-p65 using ImageJ (**B**). The data are expressed as mean ± SD. ^##^ *p* < 0.01, versus the Sham group. ** *p* < 0.01, versus the Model group.

The results of Western blot showed that the liver injury caused by HIRI and partial hepatectomy increased the expression and phosphorylation of p65. Nonetheless, ADSC and ADSC-Exo transplantation significantly inhibited the p65 expression (Figure 3 and Figure 4).

### 2.3. ADSCs-Exo Promoted Liver Regeneration

**Figure 5:**
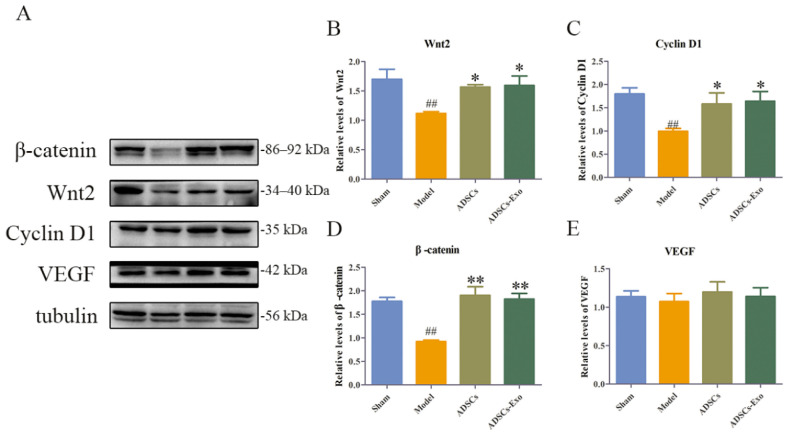
ADSCs-Exo increased the protein expression of regeneration in the liver. Representative Western blot analysis of Wnt2, Cyclin D1, β-catenin, and VEGF (**A**). Quantification of Wnt2, β-catenin, Cyclin D1, and VEGF (**B**–**E**). The data are expressed as mean ± SD. ^##^ *p* < 0.01, versus the Sham group. * *p* < 0.05 and ** *p* < 0.01, versus the Model group.

**Figure 6:**
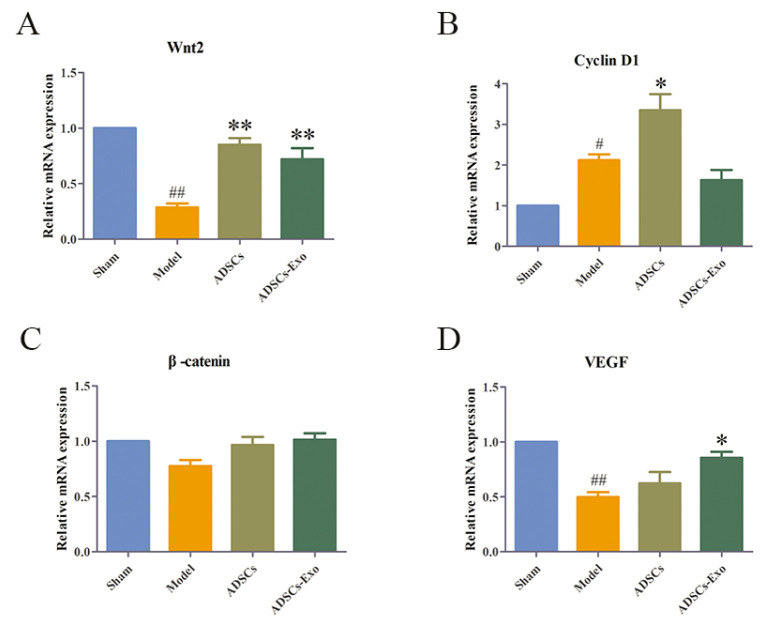
ADSCs-Exo increased the mRNA expression of regeneration in the liver. Level of Wnt2 (**A**), Cyclin D1 (**B**), β-catenin (**C**), and VEGF (**D**) in the liver in different groups. The data are expressed as mean ± SD. ^#^ *p* < 0.05 and ^##^ *p* < 0.01, versus the Sham group. * *p* < 0.05 and ** *p* < 0.01, versus the Model group.

**Figure 7:**
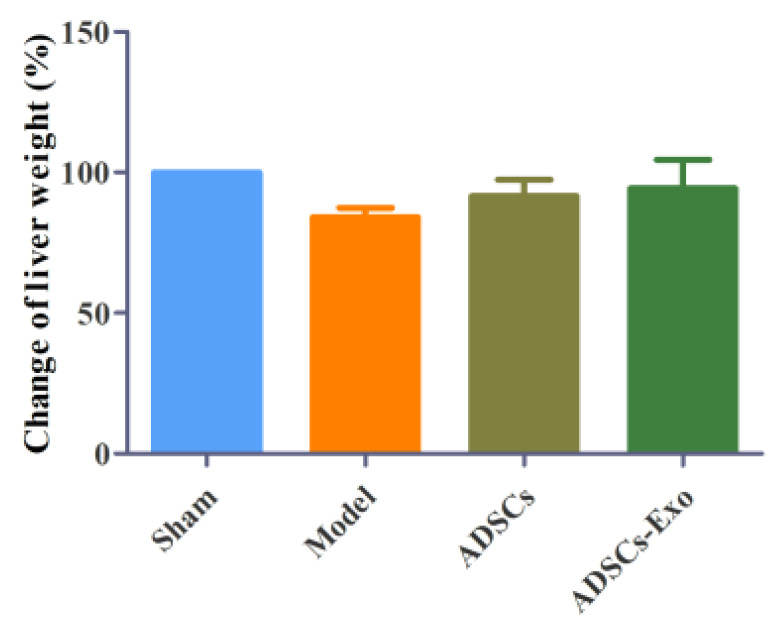
Effects of ADSCs-Exo and ADSCs on liver weight after ischemia and reperfusion combined with hepatectomy injury.

Wnt2 promotes the proliferation of hepatocytes via activating the Wnt/β-catenin pathway, which is inhibited in excessive inflammatory environments. In contrast, ADSCs and ADSCs-Exo apparently reversed the inhibition of regeneration-related factors caused by severe liver injury, such as Wnt2, β-catenin, and Cyclin D1, whereas they slightly boosted VEGF expression (Figure 5). At the genetic level, ADSCs significantly increased Wnt2 and Cyclin D1. Simultaneously, ADSCs-Exo distinctly raised the expression of Wnt2 and VEGF mRNA in the liver tissue (Figure 6). A comparison of the liver weight at 24 h of reperfusion with the original liver showed that ADSCs and ADSCs-Exo were able to promote liver regeneration(Figure 7).

## 3. Discussion

HIRI is a non-specific systemic inflammatory pathophysiological process caused by various diseases such as trauma, liver transplantation, and hepatectomy. It is a major clinical problem encountered after hepatobiliary surgery and organ transplantation, and the degree of injury determines the survival of the transplanted liver and the remaining liver. Unlike infectious immunity, it is a type of pathophysiological process that can cause systemic inflammatory dysregulation, independent of antigen [9]. The typical pathological features of HIRI include massive cell death and severe inflammatory response. These injured cells generate ROS, which causes inflammatory cell accumulation, the release of inflammatory factors such as IL-1, IL-1β, IL-18, and TNF-α, and cell pyroptosis, and further aggravates cell death, resulting in tissue injury [10].

Several studies have reported that the exosomes derived from mesenchymal stem cells (MSCs-Exo) can reduce pathophysiological reactions such as apoptosis [11,12], cellular inflammation, and oxidative stress response [13] after HIRI and promote autophagy [14] and liver regeneration [15]. However, the effect of ADSCs-Exo on pyroptosis and regeneration in HIRI combined with partial resection injury has not yet been reported in rats. In order to determine this, we injected ADSCs-Exo into rats through the tail vein immediately after surgery. We observed that pyroptosis-related gene and protein expression were significantly increased in the injured liver. However, ADSC-Exo administration inhibited pyroptosis. This is consistent with the results of previous studies that demonstrated the effect of ADSCs-Exo on liver injury [8]. It is well-known that pyroptosis is a kind of cell perforation and death that is caused by the accumulation and release of a large number of inflammatory factors by inflammatory cells. It is increasingly becoming a hot spot in research concerning ischemia–reperfusion injury, and NLRP3 is a key protein in pyroptosis-related research. Inflammasome assembly activates caspase-1, which subsequently cleaves the GSDMD protein to release the N-terminal domain (GSDMD-N), which then translocates to the plasma membrane to form a transmembrane pore. The inflammasome itself breaks down and activates pro-inflammatory cytokines, such as IL-1β and IL-18, released through the GSDMD pore, and also activates pyroptosis [16]. Yan et al. demonstrated that the exosomes derived from the human umbilical cord mesenchymal stem cells (hUMSCs-Exo) inhibit pyroptosis in skeletal muscle cells by delivering circHIPK3, thereby improving ischemic hindlimb repair. Among hUMSCs-Exo, the authors showed that circHIPK3 acts as a miR-421 molecular sponge to inhibit inflammation, thus increasing FOXO3a expression, leading to the inhibition of NLRP3 and caspase-1 [17]. Liu et al. found that the exosomes derived from the bone marrow mesenchymal stem cells (BMSCs-Exo) can inhibit inflammation and pyroptosis. Additionally, they reduce cerebral ischemia–reperfusion injury by promoting microglial polarization from M1 to M2, thereby inhibiting the expression of proteins such as NLRP3 and GSDMD [18]. Zhang et al. found that injecting the serum with exosomes derived from rats with HIRI (IRI-Exo) resulted in the pyroptosis of hippocampal and cortical neurons in normal rats. The oxidative-stress-related parameters, NLRP3, ASC, and other protein expressions were similar to those in the liver injury group. However, injecting MCC950, an inhibitor of NLRP3, could resist hepatic ischemia–reperfusion or IRI-Exo injury. Zhang’s article demonstrates, on the one hand, the characteristic that exosomes can cross the blood–brain barrier, providing a theoretical basis for treatment with exosomes in clinical practice, and on the other hand, the importance of NLRP3 in pyroptosis [19].

Knowing the critical role of NLRP3, we need to trace the upstream mechanisms that promote inflammasomes, that is, what leads to the aggregation of inflammatory factors and the reorganization of inflammasomes. According to a previous study, the NF-κB signaling pathway is one of the main mechanisms leading to HIRI [20]. NF-κB is an important factor located downstream of TLR4 in the TLR4/NF-κB pathway, which not only controls the transcription of nuclear DNA but also participates in the regulation of inflammatory responses, oxidative stress, cell differentiation, and apoptosis [21]. Many studies have confirmed that the TLR4/NF-κB signal transduction pathway plays an important role in mediating the occurrence and development of inflammation in HIRI [22,23,24]. Yu’s study showed that L-tetrahydropalmatine (L-THP) can reduce the hepatic inflammatory response, hepatocyte apoptosis, and autophagy through the ERK–NF-κB pathway, thereby effectively reducing HIRI in mice [25]. In the present study, we detected a significant elevation of NF-κB expression in the injured liver tissue in a variety of ways, while ADSCs and ADSCs-Exo could inhibit the phosphorylation of NF-κB, thereby alleviating liver injury, which is consistent with the results reported in the literature. Therefore, we can conclude that ADSCs and ADSCs-Exo could reduce cell pyroptosis and restore liver function possibly by inhibiting the NF-κB signaling pathway and alleviating the inflammatory response in the liver environment. However, it is worth mentioning that although ADSCs could reduce the expression of inflammatory response-related proteins such as NLRP3 in the liver tissues, they significantly increased the expression of GSDMD protein. This may be due to the release of IFN-β from macrophages at the injury site, which stimulates ADSCs after recruitment to the injury site, resulting in pyroptosis, so the examination of liver tissue results in the ADSC intervention group revealed elevated GSDMD expression [26]. However, this requires further validation, and in order to confirm whether ADSCs do not lead to more GSDMD expression when macrophages are lacking, rat primary hepatocytes injured by hypoxia–reoxygenation were intervened with ADSCs in vitro. Nonetheless, the current results prove the advantages and feasibility of ADSCs-Exo as a stem cell substitute in the clinical use of cell-free therapy.

Multiple studies have shown that intervening substances usually have the effect of promoting the regeneration of injured tissues or organs and inhibiting inflammation. Li et al. showed that geniposide significantly inhibits the activities of NF-κB and inflammatory factors such as TNF-α, IL-1β, NO, and PGE2, thus reducing tissue edema, inhibiting neutrophil infiltration, promoting spinal cord tissue repair and axonal myelination, and improving axonal regeneration and axoplasmic transport function at the injury site [27]. ADSCs and the conditioned media derived from ADSCs have also been reported to play a role in minipig liver injury, in promoting anti-apoptosis and regeneration [28,29]. Meanwhile, in addition to alleviating injury, promoting tissue and organ regeneration is also a research target in the clinical application of exosomes. Exosomes have long been used for the cutaneous wound healing process through mechanisms that include the inhibition of inflammation, the promotion of angiogenesis and tissue remodeling, and a reduction in scar generation [30,31]. A study describing the promotion of liver regeneration by exosomes reports that MSCs-Exo can reverse the CCL4-induced hepatic injury, most significantly manifested as the protein expression of PCNA, Cyclin D1, and Cyclin E, initiating the proliferation program of hepatocytes [32]. In addition, in the hepatectomy model, the results indicate that miR-124 carried by the hUMSC-derived exosomes promoted liver regeneration and inhibited liver injury through the expression of Foxg1 [33]. Multiple lines of evidence reveal that exosomes are important in the study of liver regeneration, and our results also show that exosomes can reverse the decreased expression of liver-regeneration-related factors such as cyclin D1 and VEGF, caused by liver injury, while we found that exosomes can activate the Wnt/β-catenin pathway to promote liver regeneration, which is consistent with previous studies. Ji et al. showed that the placenta-derived mesenchymal stem cells (PD-MSCs) can induce angiogenesis by increasing C-reactive protein (CRP) and directly play a role in the regeneration of hepatocytes by the Wnt signaling pathway [34].

## 4. Materials and Methods

### 4.1. Animals

Male Sprague Dawley rats (6–8 weeks, weighing 200–250 g) were purchased from Liaoning Changsheng Biotechnology Co., Ltd. (Shenyang, China). All the rats were controlled in a suitable feeding environment to ensure sufficient drinking water and food during feeding. All animal experiments were approved by the Institutional Animal Care and Use Committee of the Northeast Agricultural University.

### 4.2. ADSC Isolation and Expansion

The inguinal adipose tissue was removed from the rats following strict aseptic operation procedures. The tissue was washed thrice with phosphate-buffered saline (PBS) supplemented with 100 U/mL penicillin and 100 μg/mL streptomycin to remove the muscle, blood vessels, and fascia. The tissue was minced and then transferred to a 50 mL EP tube containing 0.1% collagenase type I at 37 °C for 50 min with gentle shaking. The undigested tissue was removed via centrifugation at 1200 rpm for 10 min, and digestion was terminated by the addition of a complete medium (L-DMEM, 10% fetal bovine serum, 100 U/mL penicillin, 100 μg/mL streptomycin and 2 mM L-glutamine). The suspension was filtered through a 70 μm cell strainer to remove undigested tissue fragments and again centrifuged at 1200 rpm for 10 min. The precipitated cells were resuspended in a 5 mL erythrocyte lysis buffer and centrifuged at 1200 rpm for 5 min. The cell pellet was resuspended in the complete medium, seeded in 25 cm^2^ flasks, and cultured in a humidified incubator (Galaxy 170S, Eppendorf, Hamburg, Germany) at 37 °C in 5% CO_2_ and 95% air. The ADSCs from passages 3 to 5 were washed, counted, and used for transplantation.

### 4.3. Isolation of ADSCs-Exo

The ADSCs from passages 3 to 5 were cultured in a serum-free medium for 24 h, and the exosomes were obtained via differential centrifugation. As previously described, living cells, dead cells, cell debris, and large vesicles were removed from the supernatant through centrifugation at 300× *g* for 15 min, 2000× *g* for 25 min, and 10,000× *g* for 50 min, respectively [8]. The supernatant before centrifugation at 10,000× *g* was filtered through a 0.22 μm sterile filter. The supernatant was then transferred to an ultracentrifugation tube (Beckman, CA, USA) and centrifuged at 100,000× *g* for 90 min using a Ti 50.2 rotor (Beckman, CA, USA). The obtained precipitate was fully dissolved in PBS and ultracentrifuged at 100,000× *g* for 90 min. The precipitates thus obtained are exosomes, which were resuspended in PBS and stored at −80 °C until further use.

### 4.4. Operation Methods

In this study, 24 male SD rats were randomly divided into the Sham, Model, ADSC-transplanted, and ADSC-Exo transplanted groups (n = 6 each). Then, 2 × 10^6^ ADSCs or 100 μg ADSCs-Exo were injected via the tail vein immediately after the operation. At the same time, 0.6 mL of PBS was injected into rats in the Sham or Model groups, respectively. In the Sham group, the liver lobe was slightly turned over after laparotomy, while the rats in the Model, ADSC-transplanted, and ADSC-Exo-transplanted groups underwent hepatic ischemia–reperfusion and partial hepatectomy as previously described [8].

The animals were anesthetized with isoflurane via a mask. The abdomens of the rats were shaved, disinfected, and opened with a 3 cm midline incision. After dissecting free from the surrounding ligaments of the left lateral liver lobe, the blood flow in the median and left lateral liver lobes was blocked using a non-traumatic vascular clamp, and then the lobes of the liver were seen to undergo discoloration. Partial hepatectomy (PH) was performed during ischemia by resecting the left lateral liver lobe. After clamp removal, the blood flow was restored to the ischemic liver lobes, resulting in reperfusion injury. The abdomen was closed in two layers using Safil 6–0 (Jinhuan Medical, Shanghai, China).

The median lobe and blood samples were collected at 24 h after reperfusion in each group.

### 4.5. Weight Calculations

In the Model group, the resected liver mass was weighed after hepatectomy. The initial total liver weight was calculated as follows:

$$\frac{\mathrm{Resected}\;\mathrm{liver}\;\mathrm{weight}}{30\%}\;\left(g\right).$$

At the time of sacrifice, the animals, and in the Model group, also their regenerated liver mass, were weighed. The percentage of change in the liver was calculated as follows:

$$\frac{\mathrm{Remaining}\;\mathrm{liver}\;\mathrm{weight}}{\mathrm{Initial}\;\mathrm{total}\;\mathrm{liver}\;\mathrm{weight}}\times 100\left(\%\right).$$

### 4.6. Immunofluorescence (IF)

Paraffin sections were dewaxed and rehydrated. Antigen repair was achieved using the microwave thermal repair method. The sections were blocked with bovine serum albumin (BSA) for 30 min at room temperature and then incubated overnight at 4 ℃ with primary antibodies in PBS. After three rounds of washing of PBS, the sections were incubated with secondary antibodies with PBS for 50 min at room temperature. The nuclei were subsequently counterstained with DAPI. Autofluorescence was quenched with an autofluorescence quencher (Servicebio, Beijing, China). Finally, the anti-fluorescence quencher (Servicebio, Beijing, China) was used to seal the sections.

### 4.7. ELISA

The levels of IL-18 in the serum were measured using specific ELISA kits according to the manufacturer’s instructions (Jingmei Biotechnology, Yancheng, China).

### 4.8. Real-Time Quantitative PCR (RT-qPCR) Analysis of mRNA

The total RNA was extracted from the liver tissues using a TRIzol reagent (Invitrogen, Shanghai, China) and reverse-transcribed to cDNA using a PrimeScript^TM^ RT reagent kit with gDNA Eraser (TaKaRa, Beijing, China). RT-qPCR was performed using Taq SYBRGreen qPCR Mix (Innovagene, Changsha, Hunan, China) on LightCycler 480 (Roche, Basel, Switzerland). The reaction mixture consisted of 2 μL cDNA, 1 μL each of forward and reverse primers, 6 μL dH2O, and 10 μL fluorescence dye. The relative expression of mRNA was calculated using the 2^−ΔΔCt^ method. The primers were purchased from Tsingke Biotechnology Co., Ltd. (Beijing, China), and the sequences are listed in Table 1.

### 4.9. Western Blot

The total protein was extracted from the liver tissues using a Total Protein Extraction kit (Beyotime, Shanghai, China) and quantified using a BCA kit (Beyotime, Shanghai, China). Equal amounts of protein per sample (30 μg) were separated on a 10% SDS–PAGE and electroblotted onto nitrocellulose membranes (Pall Corporation, Port Washington, NY, USA). After blocking with 5% non-fat milk for 2 h, the blots were incubated overnight with anti-VEGF (1:2000; Wanleibio, Shenyang, Liaoning, China), anti-Cyclin D1 (1:1000; Wanleibio, Shenyang, Liaoning, China), anti-NLRP3 (1:2000; Bioss, Beijing, China), anti-Wnt2 (1:1000; Abcam, Waltham, MA, USA), anti-β-catenin (1:10,000; Abcam, Waltham, MA, USA), anti-P-P65 (1:500; Santa, Dallas, TX, USA), anti-P65 (1:3000; Proteintech, Wuhan, Hubei, China), anti-caspase-1 (1:500; Santa, Dallas, TX, USA), anti-ASC (1:500; Santa, Dallas, TX, USA), anti-GSDMD (1:2000; Affinity, Liyang, Jiangsu, China), and anti-β-tubulin (1:1000; Wanleibio, Shenyang, Liaoning, China) primary antibodies at 4 ℃. The blots were washed with TBST and incubated with HRP-conjugated anti-IgG for 2 h. The positive bands were detected using an enhanced ECL reagent (Meilunbio, Dalian, Liaoning, China) on an AI600 System (GE Healthcare, Pollards Wood, UK). The relative protein expression was quantified using ImageJ software and normalized against the β-tubulin control.

### 4.10. Statistical Analysis

SPSS 21.0 software was used for data analysis. The data are expressed as mean ± standard deviation and compared using a one-way analysis of variance (ANOVA). A *p*-value < 0.05 was considered to be statistically significant.

## 5. Conclusions

**Figure 8:**
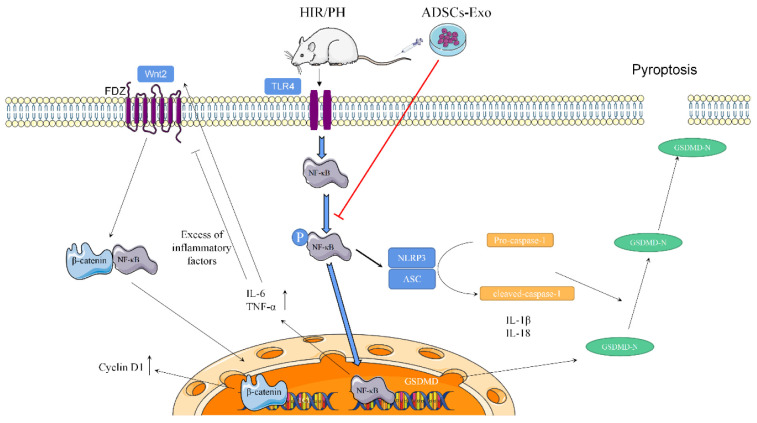
The mechanism of ADSCs-Exo alleviates pyroptosis and promotes tissue regeneration in the liver, which had ischemia–reperfusion and partial resection injury. The results show that ADSCs-Exo inhibits the NF-κB pathway and activates the Wnt/β-catenin signal pathway. The mechanism may be like that in Figure 8. Liver injury leads to an inflammatory response that will restrain hepatocyte proliferation if it is too serious. After transplanting ADSCs-Exo, the phosphorylation of NF-κB is inhibited and fails to stimulate the production of inflammation and the activation of GSDMD. On the other hand, ADSCs-Exo can activate the Wnt/β-catenin pathway, and β-catenin translocates into the nucleus, increasing the expression of Cyclin D1 and thereby promoting cell proliferation. Ultimately, ADSCs-Exo play a role in mitigating cell damage and promoting tissue regeneration and functional recovery.

In summary, after hepatic ischemia–reperfusion combined with partial resection injury in rats, ADSC-Exo administration inhibits the pyroptosis resulting from inflammatory responses, reduces liver injury, and promotes liver regeneration; related mechanisms may be through the inhibition of the NF-κB pathway and the activation of the Wnt/β-catenin pathway (Figure 8). It is worth noting that ADSCs-Exo outperform ADSCs in inhibiting pyroptosis, providing a theoretical foundation for the use of exosomes in clinical studies for the treatment of liver injuries.

## Figures and Tables

**Table 1:** Gene-specific primers used in the qPCR.

Gene	Primer Sequences (5′ to 3′)
NF-κB F	GATCGCCACCGGATTGAAGA
NF-κB R	CTCGGGAAGGCACAGCAATA
GSDMD F	CTGGGAGATCATGCAACGTG
GSDMD R	TCACCATCTTCTTCCGGCTT
NLRP3 F	ATTACCCGCCCGAGAAAGG
NLRP3 R	CATGAGTGTGGCTAGATCCAAG
IL-18 F	CGACCGAACAGCCAACGAATCC
IL-18 R	GTCACAGCCAGTCCTCTTACTTCAC
pro-caspase-1 F	AAACACCCACTCGTACACGTCTTG
pro-caspase-1 R	AGGTCAACATCAGCTCCGACTCTC
ASC F	ATGGTTTGCTGGATGCTCTGTATGG
ASC R	AAGGAACAAGTTCTTGCAGGTCAGG
Cyclin D1 F	GAGGCGGATGAGAACAAGCAGATC
Cyclin D1 R	GGAGGGTGGGTTGGAAATGAACTTC
VEGF F	CACCAAAGCCAGCACATAGGAGAG
VEGF R	CTGCGGATCTTGGACAAACAAATGC
Wnt2 F	TTCTGAAGCTGGAGTGCAAGTGTC
Wnt2 R	TCATATCGCCTCCTCAGGTAGTCAC
β-catenin F	ACAAGCCACAGGACTACAAGAAACG
β-catenin R	TCAGCAGTCTCATTCCAAGCCATTG
β-actin F	TGTCACCAACTGGGACGATA
β-actin R	GGGGTGTTGAAGGTCTCAAA

## Data Availability

The data used to support the findings of this study are available from the corresponding author upon request.

## Abbreviations

HIRI	Hepatic ischemia–reperfusion injury
ADSCs	Adipose-derived stem cells
ADSCs-Exo	Exosomes derived from adipose-derived mesenchymal stem cells
NLRP3	NOD-, LRR-, and pyrin-domain-containing protein 3
ASC	Apoptosis-associated speck-like protein containing a card
caspase-1	Cysteinyl aspartate proteases-1
GSDMD	Gasdermin-D; IL-18: interleukin-18
NF-κB	Nuclear transcription factor-κB
Wnt2	Wnt family member 2
VEGF	Vascular endothelial growth factor
